# Is Vaginal Breech Delivery Still a Safe Option?

**DOI:** 10.1055/s-0040-1713804

**Published:** 2020-11-30

**Authors:** Maria Pulido Valente, Maria Carvalho Afonso, Nuno Clode

**Affiliations:** 1 Department of Obstetrics, Gynecology and Reproductive Medicine, Hospital de Santa Maria, Centro Hospitalar Universitário Lisboa Norte, Lisboa, Portugal

**Keywords:** breech presentation, vaginal delivery, maternal morbidity, neonatal morbidity, apresentação pélvica, parto vaginal, morbidade maternal, morbidade perinatal

## Abstract

**Objective** To determine whether there was any difference in neonatal and maternal outcomes between breech vaginal delivery and cephalic vaginal delivery.

**Methods** A retrospective, case-control study was conducted between January 2015 and December 2017 in a Portuguese hospital. A total of 26 cases of breech vaginal delivery were considered eligible and 52 pregnant women formed the control group.

**Results** Induced labor was more frequent in the breech vaginal delivery group (46% versus 21%, *p* = 0.022). Episiotomy was more common in the breech vaginal delivery group (80% versus 52%, *p* = 0.014), and one woman had a 3 ^rd^ degree perineal laceration. Newborns in the study group had a lower birthweight (2,805 g versus 3,177 g, *p* < 0.001). There was no significant difference in the neonatal outcomes.

**Conclusion** The present study showed that breech vaginal delivery at term compared with cephalic presentation was not associated with significant differences in neonatal and maternal morbidity. It also suggests that breech vaginal delivery remains a safe option under strict selection criteria and in the presence of an experienced obstetrician.

## Resumo

**Objetivo** Avaliar se existem diferenças nos desfechos perinatal e materno entre os partos pélvicos vaginais e eutócicos.

**Métodos** Realizou-se um estudo retrospectivo, caso controle, entre janeiro de 2015 e dezembro de 2017 em um hospital terciário em Portugal. Foram incluídos 26 casos de parto pélvico vaginal e o grupo controle foi formado por 52 grávidas.

**Resultados** A indução do trabalho de parto ocorreu com mais frequência no grupo do parto pélvico vaginal (46% *versus* 21%, *p* = 0.022), bem como a realização de episiotomia (80% *versus* 52%, *p* = 0.014). Verificou-se um caso de laceração perineal de 3° grau. Os recém-nascidos do grupo de estudo apresentaram um peso inferior ao nascimento (2.805 g *versus* 3.177 g, *p* < 0.001). Em relação aos desfechos perinatais, não se observaram diferenças estatisticamente significativas.

**Conclusão** O presente estudo mostrou que o parto pélvico vaginal no termo quando comparado com o parto eutócico não se associou a diferenças estatisticamente significativas na morbidade perinatal e materna, e sugere ainda que o parto pélvico vaginal parece ser uma opção segura em casos rigorosamente selecionados e na presença de um obstetra experiente.

## Introduction

Breech presentation occurs in between 3 and 4% of term deliveries. 1 The best route of delivery is still controversial, but the safety of vaginal breech delivery is the main concern. Over the past 20 years, several studies have been published to understand which is the safest mode of delivery, not only for the fetus but also for the mother and subsequent pregnancies. Since the publication of the Term Breech Trial (TBT) in 2000, planned cesarean section was routinely recommended for the delivery of the fetus in the breech presentation at term. The TBT showed reduced perinatal mortality and serious neonatal morbidity in the planned caesarean section group (1.6%) compared with the planned vaginal birth group (5%) (relative risk [RR] 0.33, *p* < 0.0001). 1 However, there was no difference in the long-term perinatal morbidity between the two groups nor in serious maternal morbidity. 2 Furthermore, the TBT had several weaknesses that prevent the generalization of the results. The analysis by Glezerman revealed some of these: violation of inclusion criteria such as inclusion of fetus with hyperextended head or > 4,000 g in the planned vaginal delivery; substantially different levels of standard of care between the participating centers and, equally important, > 20% of the planned vaginal deliveries were performed by a less skilled obstetrician. 3 On the other hand, the PREsentation et MODe d'Accouchement (PREMODA) study, a prospective observational multicenter study conducted in 174 centers in France and Belgium including 8,105 singleton breech fetuses at term, showed that a composite outcome of fetal/neonatal mortality or serious neonatal morbidity was not significantly different for planned vaginal versus planned cesarean delivery (1.60% versus 1.45%, odds ratio [OR] 1.10, 95% confidence interval [CI]: 0.75–1.61). 4 Moreover, cesarean delivery was associated with several maternal morbidities, such as hemorrhage that requires hysterectomy or transfusion, uterine rupture, anesthetic complications, shock, cardiac arrest, acute renal failure, assisted ventilation, venous thromboembolism, major infection, or in-hospital wound disruption or hematoma; and also long-term risks, especially those associated with subsequent pregnancies, such as placental disorders. 5 Most of the studies published, either retrospective or prospective, compare vaginal breech delivery with planned or intrapartum cesarean delivery. However, it is well-known that breech presentation, regardless the mode of delivery, is itself associated to worse outcomes. 6 The present study was designed to determine whether there was any difference in neonatal and maternal outcomes between breech vaginal delivery and cephalic vaginal delivery in a selected population.

## Methods

A retrospective, case-control study was conducted between January 2015 and December 2017 in a Portuguese hospital (Hospital de Santa Maria, Centro Hospitalar Universtário Lisboa Norte, Lisboa, Portugal). This is a tertiary hospital with a neonatal intensive care unit (NICU) and ∼ 2,500 deliveries per year, where breech vaginal delivery is an option for those who desire it. The inclusion criteria were singleton term (≥ 37 weeks), absence of major fetal anomaly, breech presentation (frank or incomplete) without hyperextended neck, estimated fetal weight between 2,000 and 3,500 g, absence of fetal or maternal contraindication for vaginal delivery, and no history of previous uterine scar. For each pregnant woman in the study group, two pregnant women with the same parity and gestational age were selected for control, who had a cephalic vaginal delivery immediately before and after the index case, according to the delivery suite database. All women with fetuses with breech presentation had an informed consent for vaginal breech delivery. Electronic fetal monitoring was performed throughout the entire labor as well as regional analgesia whenever requested, and an experienced obstetrician was present in all breech deliveries. Induction of labor was done with misoprostol 25 μg vaginally every 4 hours until a maximum dose of 125 μg; labor augmentation was done with oxytocin with a rate perfusion starting from 2.5 mUI/minute with increases of 2.5 mUI/minute every 15 minutes until 3 to 4 contractions every 10 minutes were reached. Cases were identified using the database of the hospital. The maternal and neonatal medical records were reviewed, and data was obtained and inserted in a standardized data sheet. The primary outcomes were defined as maternal (3 ^rd^ and 4 ^th^ degree laceration, cervical tear, postpartum hemorrhage > 1,500 ml, postpartum fever) and neonatal morbidity (birth trauma; 5-minute Apgar score < 7; fetal acidemia; admission to NICU for > 4 days) and mortality. We analyzed demographic and obstetrics characteristics such as maternal age, ethnicity and parity. Concerning the current pregnancy, variables included gestational age at delivery, fetal presentation, need for induction of labor, regional analgesia, need of episiotomy, perineal trauma and Piper forceps application. Birthweight and Apgar score were registered. Birth trauma was defined as subdural hematoma, intracerebral or intraventricular hemorrhage, spinal-cord injury, basal skull fracture, clinically significant genital injury, brachial plexus injury, humerus or clavicle fracture. The study protocol was approved by the local ethics committee. The groups were compared with the Mann-Whitney test for continuous variables and with the χ ^2^ test for categorical variables. A *p-value* < 0.05 was considered statistically significant. Statistical comparisons were performed with IBM SPSS Statistics for Windows, version 23.0 (IBM Corp., Armonk, NY, USA).

## Results

**Fig. 1:**
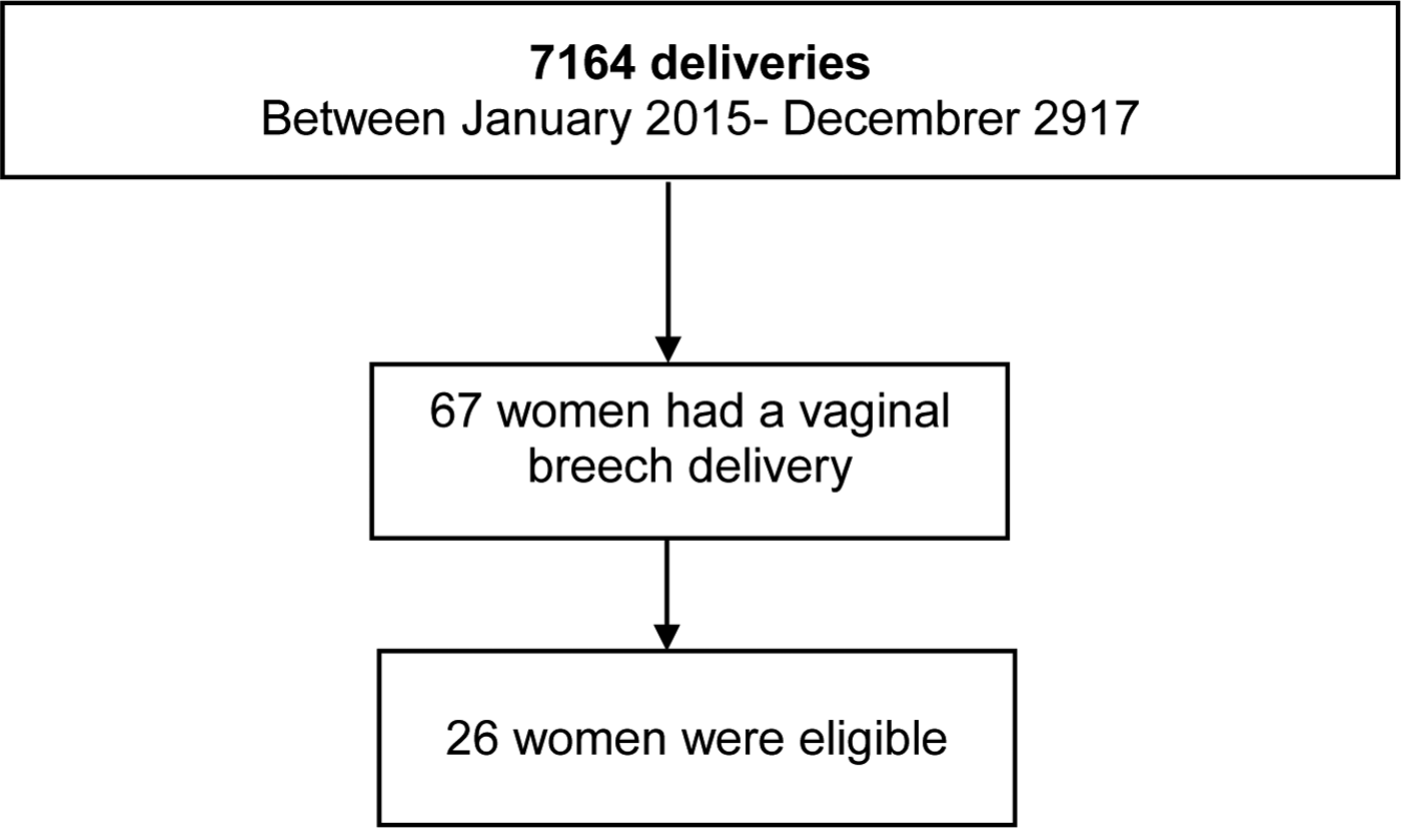
Flowchart summarizing inclusion of women in the present study.

From January 2015 through December 2017, there were a total of 7,164 deliveries, of which 67 were breech vaginal deliveries. During this period, 26 cases (39%) of breech vaginal deliveries were considered eligible for the present study ( Fig. 1 ). A total of 52 pregnant women formed the control group.

The demographic characteristics studied were similar in both groups ( Table 1 ), as well as gestational age at birth. Induced labor was more frequent in the breech vaginal delivery group (46% versus 21%, *p* = 0.022). Regional analgesia was performed in 93% of the deliveries of the present study and there was no difference among the groups (96% versus 88%, *p* = 0.262) ( Table 1 ).

**Table 1:** Demographic characteristics

	Vaginal Breech Delivery ( *n* = 26)	Cephalic Delivery ( *n* = 52)	*p-value*
Age a (years old)	33	31	0.067
Parity			
Nulliparous	11 (42%)	22 (42%)	1
Multiparous	15 (58%)	30 (58%)	
Race			
Caucasian	22 (85%)	39 (75%)	0.332
Other	4 (15%)	13 (25%)	
Gestational age a (weeks)	39	39	1

a median.

Tables 2 and 3 report delivery characteristics and maternal and neonatal outcomes. Concerning the delivery, episiotomy was more common in the breech vaginal delivery group (80% versus 52%, *p* = 0.014), and just one woman had a 3 ^rd^ degree perineal laceration. Newborns in the study group had a lower birthweight compared with the cephalic vaginal delivery group (2,805 g versus 3,177 g, *p* < 0.001). Piper forceps was used in just 3 (12%) of the breech vaginal deliveries, but no other maneuvers were needed. Regarding the neonatal outcomes, there was no significant difference between the two groups. In the breech vaginal delivery group, there was one case of 5-minute Apgar score < 7 and one neonate was admitted to the NICU due to a postpartum diagnosis of cleft lip and palate. There were no reported maternal or neonatal deaths.

**Table 2:** Delivery characteristics

	Vaginal Breech Delivery ( *n* = 26)	Cephalic Delivery ( *n* = 52)	*p-value*
Induced labor	12 (46%)	11 (21%)	0.022
Epidural anesthesia	25 (96%)	46 (88%)	0.262
Episiotomy	21 (80%)	27 (52%)	0.014
Piper Forceps	3 (12%)	0	0.012
Birthweight a (grams)	2,805	3,177	<0.001

a median.

**Table 3:** Neonatal and maternal outcomes

	Vaginal Breech Delivery ( *n* = 26)	Cephalic Delivery ( *n* = 52)	*p-value*
5-minute Apgar score < 7	1 (3.8%)	0	0.155
Fetal acidemia	0	0	
NICU admission	1 (3.8%)	0	0.155
Birth trauma	0	0	
3 ^rd^ and 4 ^th^ grade laceration	1 (3.8%)	0	0.155
Postpartum hemorrhage	0	3 (5.7%)	0.212
Postpartum fever (%)	0	0	

Abbreviation: NICU, neonatal intensive care unit.

## Discussion

Our study shows that vaginal delivery for breech presentation seems as safe as for cephalic presentations in a selected population and if a trained obstetrician is present in breech delivery.

We found no significant difference in the neonatal and maternal morbidity in the vaginal delivery, regardless of fetal presentation, which can be explained by several reasons. First, due to only a highly selected population being allowed for a trial of labor. This is consistent with previous studies that did not find a significant risk associated with planned vaginal delivery compared with planned cesarean in a selected population. 4 7 Although a recent meta-analysis that included observational studies has shown that there is an increased relative risk of perinatal mortality and morbidity in vaginal breech delivery compared with cesarean section, the absolute risk was very small (for example, perinatal death 0.3% versus 0.05%, respectively). 8

Second, labor management was done by an experienced obstetrician in breech delivery. Roughly 21.4% of the vaginal deliveries in the TBT were assisted by obstetricians in training or a licensed midwife, without any supervision, and this could be one of the explanations for a worse neonatal outcome. Furthermore, several international societies have advocated that a trial of labor for breech presentation is possible with appropriate case selection, management according to a strict protocol and the availability of skilled attendants. 9 10

About 46% of women with a breech presentation in our study had an induced labor, to ensure an experienced obstetrician present at the time of delivery. Until now, there is no international consensus about labor induction of breech presentation. The American guidelines do not provide any recommendation about labor induction, and the Royal College of Obstetricians and Gynaecologists (RCOG) in the UK advises that women should be informed that labor induction is not usually recommended. 9 10 However, the Collège National des Gynécologues Obstétriciens Français (CNGOF) suggest that labor induction can be an option if the optimal obstetrical conditions are present. 11

Only a few studies have evaluated labor induction of breech presentation, and their results are quite reassuring. One of these studies compares women with induced labor with women with spontaneous onset of labor. There was no significant difference in cesarean rate, neonatal or maternal morbidity (1.69, OR = 0.71–4.04; 0.52, OR = 0.12–2.35; and 0.73, OR = 0.21–2.51, respectively). 12 A secondary analysis of the PREMODA study also demonstrated that induction of labor for breech presentation did not appear to increase neonatal mortality or severe neonatal morbidity compared with planned caesarean delivery (1.4 versus 1.2, *p* = 0.75). 13 Therefore, it seems reasonable to promote induction of labor in a term breech presentation in order to have a skilled obstetrician present during the delivery.

In our cohort, the birthweight of breech fetuses was lower (median 2,805 g versus 3,177 g, *p* < 0.001) compared with cephalic vaginal deliveries. Such finding can be explained by the strict selection criteria applied before a trial of vaginal breech delivery. Only fetuses with estimated fetal weight between 2,000 and 3,500 g were proposed for a trial of labor.

The main strength of our study is its original subject. To our knowledge, the present study is the first that compares vaginal delivery morbidity according to the fetal presentation. Until now, all the studies compared breech vaginal delivery with planned cesarean section. We believe that this comparison does not allow understanding the effect of vaginal delivery in fetal and maternal morbidity. In fact, vaginal cephalic delivery per se is associated to an increased risk of birth trauma, as shoulder dystocia, related-brachial plexus injuries, subgaleal hemorrhage and cephalohaematoma, and comparing to cesarean section the overall perinatal morbidity is higher.

Nevertheless, international societies do not consider cesarean section the standard of care for cephalic babies.

Another strength is the study design and selection of the control group that had the same gestational age and parity of the study group, which diminishes the possibility of bias in the morbidity outcome.

The limitations are mostly related to the retrospective nature of the study and the size of the study population. When comparing our data with published ones, the neonatal morbidity of vaginal breech delivery is slightly higher for birth trauma (3.8% versus 1.8–0.7%), 5-minute Apgar score < 7 (3.8% versus 1.48–3.0%) and NICU admission (3.8% versus 2.2–3%), but there was no case of fetal acidemia. 1 4 7 8 This finding can be easily explained due to the small sample. Furthermore, the baby admitted at the NICU was not related with the mode of delivery but due to a malformation diagnosis at the time of delivery.

## Conclusion

In conclusion, the present study showed that breech vaginal delivery at term compared with cephalic presentation was not associated with significant differences in neonatal and maternal morbidity. It also suggests that breech vaginal delivery remains a safe option under strict selection criteria and in the presence of an experienced obstetrician. These facts should be taken into account when counseling women for vaginal breech delivery, as a primary cesarean section is associated with a higher maternal morbidity, especially for future pregnancies.
